# Comparison of Primary Human Osteoblast-like Cells and hFOB 1.19 Cells: Contrasting Effects of Proinflammatory Cytokines

**DOI:** 10.3390/cells14161264

**Published:** 2025-08-15

**Authors:** Juliana Franziska Bousch, Christoph Beyersdorf, Katharina Schultz, Matthis Schnitker, Christoph Viktor Suschek, Uwe Maus

**Affiliations:** Department for Orthopedics and Trauma Surgery at the University Hospital, Medical Faculty, Heinrich Heine University Düsseldorf, 40225 Düsseldorf, Germany; christoph.beyersdorf@med.uni-duesseldorf.de (C.B.); katharina.schultz@med.uni-duesseldorf.de (K.S.); matthis.schnitker@med.uni-duesseldorf.de (M.S.); suschek@hhu.de (C.V.S.); uwe.maus@med.uni-duesseldorf.de (U.M.)

**Keywords:** osteogenesis, osteoblast, proinflammatory cytokines, inflammation, hFOB 1.19, osteoimmunology

## Abstract

Proinflammatory cytokines such as IL-1β, IL-6, and TNF-α are key mediators of inflammatory bone loss and are commonly described as inhibitors of osteoblast function. However, their effects on osteogenesis remain controversial, likely due to the differences in the cell models and experimental settings in in vitro studies. We recently showed that these cytokines significantly enhanced the mineralization of primary human osteoblast-like cells (OBs). Here, we provide the first analysis of cytokine effects on the osteogenesis of the widely used human osteoblastic cell line hFOB 1.19 and compare them to primary OBs. Unexpectedly, all three cytokines significantly inhibited mineralization in hFOB 1.19 cells without affecting the proliferation. IL-1β and TNF-α also suppressed ALP activity, whereas IL-6 acted ALP-independent but increased the osteogenic marker expression despite the reduced mineralization, indicating a possible uncoupled differentiation and mineralization. Morphological and transcriptional analyses indicated that hFOB 1.19 cells represent an earlier osteogenic differentiation stage, while primary OBs show phenotypic heterogeneity and donor-dependent expression profiles. These data demonstrate that proinflammatory cytokines can have severely different effects on the osteogenesis of different cell models, supported by the highly contradictory findings reported in the literature. Nevertheless, elucidating the mechanisms underlying the inhibition of osteogenesis in hFOB 1.19 cells may provide important insights into the cell model and differentiation-stage-specific cytokine effects.

## 1. Introduction

Proinflammatory cytokines such as IL-1β, IL-6, and TNF-α are key mediators of pathological bone loss in conditions like osteoporosis and rheumatoid arthritis, primarily due to their well-described role in promoting osteoclastogenesis and bone resorption [1,2]. At the same time, they are widely regarded as inhibitors of osteoblast function and bone formation [3,4]. However, several studies have reported the stimulatory effects of these cytokines on the osteogenic differentiation of human mesenchymal stem cells (MSCs), suggesting a more complex regulatory mechanism [5,6,7]. Extending these findings, we recently demonstrated for the first time that proinflammatory cytokines also enhance osteogenesis in primary human osteoblast-like cells (OBs), a clinically relevant model for mature osteoblast function [8].

Osteoblasts originate from MSCs and are responsible for bone formation by producing and mineralizing an extracellular matrix. Their differentiation proceeds through distinct stages, which can be broadly delineated by the expression of stage-specific markers [9]. The mRNA expression levels of *alkaline phosphatase* (*Alp*), *collagen type I* (*Col1*), and *Runt-related transcription factor 2* (*Runx2*) are considered markers of early osteogenic differentiation, whereas *osteocalcin* (*Ocn*) and *osteopontin* (*Opn*) are indicative of the late phase of osteogenesis [10,11]. Osteoblastogenesis is tightly regulated by a complex network of proteins and cell–cell communications, with various signaling pathways involved [12].

The reasons for the conflicting findings in the literature remain unclear and require further investigation, as understanding osteoblast responses to acute and chronic inflammation may help identify new therapeutic strategies for inflammatory bone loss. A likely contributing factor is the variability in the cellular models used in in vitro studies. Although primary human osteoblasts provide a physiologically relevant system and a model for studying osteoblast function under disease-specific conditions, their use is limited by strong inter-donor variability due to donor-specific factors, affecting their proliferation and differentiation capacity [13,14]. As a result, many studies rely on other cell models, ranging from human or rodent MSCs, rodent primary OBs, or various cell lines like MC3T3-E1 or MG-63 [15,16,17,18,19].

A promising alternative is the human fetal osteoblast cell line hFOB 1.19, which exhibits typical osteoblastic properties and is frequently used in in vitro bone research due to its robust proliferative and osteogenic potential [20]. This cell line was established from cells of the limb tissue obtained after a spontaneous miscarriage and immortalized using a temperature-sensitive SV40 large T antigen mutant [21]. The colony with the highest ALP activity, hFOB 1.19, was selected. It was later shown to express osteogenic markers, form bone tissue in vitro and in vivo, and display minimal karyotypic abnormalities compared to MG-63 cells [22]. Due to its human, non-tumorigenic origin, the cell line has higher translational relevance for clinical questions compared to alternative osteoblast cell lines (e.g., the widely used MC3T3-E1 cells). To date, no studies have investigated the effects of proinflammatory cytokines on the osteogenesis of hFOB 1.19 cells.

In this study, we compare hFOB 1.19 cells to primary human OBs concerning their osteogenic differentiation capacity and their response to proinflammatory cytokines. By analyzing the cell morphology, cytoskeletal organization, matrix mineralization, and osteogenic marker expression, we aim to evaluate whether hFOB 1.19 cells replicate the cellular responses to inflammatory stimuli observed in primary OBs. This comparative approach is essential for assessing the translational value of hFOB 1.19 cells in inflammation-related bone research.

## 2. Materials and Methods

### 2.1. Materials

Cell culture materials were obtained from Sarstedt AG & Co. KG (Nümbrecht, Germany), and chemicals were purchased from Sigma-Aldrich (St. Louis, MO, USA). Other materials and reagents were obtained from Merck KGaA (Darmstadt, Germany), unless stated otherwise.

### 2.2. Bone Material, Ethics Approval, and Patient Information

Primary human osteoblast-like cells (OBs) were isolated from femoral heads obtained after arthroplasty at the Department of Orthopedics and Trauma Surgery at the University Hospital Düsseldorf (Düsseldorf, Germany) due to osteoarthritis or fracture, approved by the local Research Ethics Committee (study No. 5585R). Patients (9 females, 6 males; mean age 76.6 ± 9.8 years) provided written consent. Cells from patients with healthy bone density (T-score > 1.0; *n* = 5; 1 female, 4 male; mean age 74 ± 10.6 years) were used for qPCR analyses, while pooled OBs from patients without DXA data (5 females, 5 males; mean age 77 ± 9.4 years) were used for morphological analyses [23].

### 2.3. Isolation and Culture of Primary Human Osteoblasts

Prior to isolation, femoral heads were stored in PBS with 1% Pen/Strep. The procedure for isolating the OBs by collagenase type IV (787.5 U/mL; Gibco^®^ by Life Technologies™, Carlsbad, CA, USA) digestion has been described in a previous study [8]. To induce osteogenesis, the cells were incubated with osteogenesis induction medium (OB-OIM) consisting of DMEM high glucose, 10% FCS and 1% Pen/Strep, as well as the osteogenesis-inducing additives 50 µM L-ascorbic acid 2-phosphate, 10 mM β-glycerophosphate disodium salt hydrate, and 500 nM dexamethasone [24]. Cells were maintained in a humidified chamber at 37 °C with 5% CO_2_. Details on the well plates used and the number of cells seeded for each experiment are provided in Table S2.

### 2.4. Cell Culture with hFOB 1.19 Cells

The hFOB 1.19 cell line (ATCC^®^; Manassas, VA, USA) was maintained in growth medium (Ham’s F12/DMEM with 2.5 L-glutamine without phenol red (Gibco^®^ by Life Technologies™, Carlsbad, CA, USA), 10% FBS, 0.3 mg/mL Geneticin) [21]. The cells were passaged 2–3 times per week. For osteogenesis induction, 50 µM L-ascorbic acid 2-phosphate, 10 mM β-glycerophosphate disodium salt hydrate, and 500 nM dexamethasone were added to growth medium (hFOB-OIM). The cells were maintained in a humidified chamber at 34 °C with 5% CO_2_. Details on the well plates used and the number of cells seeded for each experiment are provided in Table S2.

### 2.5. Toluidine Blue, Oil Red O, and Phalloidin Staining

hFOB 1.19 cells and OBs were seeded at 50% confluency and fixed with 4% paraformaldehyde (PFA) for 15 min at room temperature (RT). For combined Oil Red O and toluidine blue staining, cells were first incubated with 0.3% Oil Red O solution for 15 min, followed by thorough washing with ddH_2_O. Subsequently, cells were incubated with 0.3% toluidine blue solution for 1–3 min, washed again with ddH_2_O, and imaged by bright-field microscopy.

For phalloidin staining, cells were permeabilized with 0.1% Triton-X-100 for 10 min, incubated with Alexa Fluor Plus 647-phalloidin (Invitrogen, Carlsbad, CA, USA) for 60 min at RT, and counterstained with DAPI (1:1000) for 10 min. Fluorescence and bright-field images were taken using a Keyence BZ-X800 microscope (Keyence, Osaka, Japan) using a 20× objective.

### 2.6. Alizarin Red S Staining

hFOB 1.19 cells and OBs were seeded at 100% confluency and treated with the recombinant human cytokines IL-1β (1 Unit/mL = 1 pg/mL), IL-6 (1 Unit/mL = 100 pg/mL), and TNF-α (1 Unit/mL = 50 pg/mL) (Peprotech, Hamburg, Germany). The cytokine concentrations (3.125–250 Units/mL) were chosen to span the pathological range reported in synovial fluid of rheumatoid arthritis patients, and to ensure comparability of biological activity across cytokines by using activity-based units [25,26].

Matrix mineralization was assessed using alizarin red S staining. For staining, the cells were fixed with 4% PFA for 15 min, and 0.5% alizarin red S staining solution was added for 20 min at 37 °C. After thoroughly washing with ddH_2_O, the stained cell cultures were microscopically observed under a transmitted light microscope with a 10× objective. By dissolving the staining with 500 µL of 10% (1 hexadecyl) pyridinium chloride monohydrate (CPC; Thermo Fisher Scientific GmbH, Karlsruhe, Germany), the staining was quantified photometrically at 600 nm.

### 2.7. MTT Assay

Cell number was quantified by Dimethylthiazolyl Blue Tetrazolium Bromide (MTT) assays. hFOB 1.19 cells were seeded at 100% confluency and treated with and without cytokines for 14 and 21 days in GM or OIM. 0.5 mg/mL MTT, diluted in hFOB-GM, was added to the cells for 2 h of incubation at 34 °C. The produced formazan was redissolved with dimethyl sulfoxide (DMSO) and measured photometrically at 540 nm [27].

### 2.8. Extracellular ALP Activity

To quantify the alkaline phosphatase (ALP) activity of hFOB 1.19 cells, they were seeded at 100% confluency and incubated with and without cytokines for 14 and 21 days. After washing with 1 mL PBS, the ALP substrate p-nitrophenyl phosphate was added and incubated for 10 min at 34 °C. The supernatant was transferred to a 96-well plate for measurement with two technical replicates and the absorption was determined photometrically at 405 nm [28]. The ALP activity values were normalized on the cell number on the corresponding day, measured by MTT assay.

### 2.9. Cell Proliferation Rate by BrdU Assay

Cell proliferation was assessed using the colorimetric bromodeoxyuridine (BrdU) kit (Roche Holding AG, Basel, Switzerland) according to the manufacturer’s protocol. hFOB 1.19 cells were seeded at 60% confluency and, after 48 h of rest, treated with hFOB-GM or -OIM with and without cytokines with the addition of 10 µM BrdU labeling solution. After 24 h of incubation at 34 °C and 5% CO_2_, the cells were fixed with FixDenat^™^ for 30 min at RT. The cells were then incubated with anti-BrdU-POD working solution for 90 min at RT. After 15 min of incubation with TMB substrate, its conversion was stopped by the addition of 1 M sulphuric acid, followed by detection in the photometer at 450 nm.

### 2.10. Real-Time qPCR

To quantify the mRNA expression of osteogenic differentiation markers, the hFOB 1.19 cells and OBs were seeded at 100% confluency. Osteogenesis was induced by adding OIM for 7, 14, 21, 28, and 35 days with and without cytokines for 21 days. For isolation of total cellular RNA, cells were lysed using the RNeasy^®^ Mini Kits (QIAGEN N.V., Hilden, Germany). The RNA was converted into cDNA using Omniscript^®^ RT Kits (QIAGEN N.V., Hilden, Germany). RT-qPCR was performed using Power SYBR^®^ Green PCR Master Mix (Thermo Fisher Scientific GmbH, Karlsruhe, Germany) with 1 ng of cDNA to measure the expression of *Alp*, *Col1α2*, and *Ocn* and 10 ng of cDNA for *Runx2* and *Opn* per 25 µL PCR reaction using a 7300 Real-Time PCR System (Applied Biosystems, Waltham, MA, USA). Reference genes were selected due to the stable expression under given conditions by using the RefFinder tool [29]. Therefore, *ribosomal protein lateral stalk subunit P0* (*Rplp0*) was used as the reference gene for hFOB 1.19 cells and *transferrin receptor protein 1* (*Tfrc*) for OBs. For each sample, three technical replicates were analyzed. Replicates with a standard deviation of >0.5 Ct were excluded in the statistical analysis, in accordance with established recommendations [30]. The 2^−ΔΔCt^ method was used to calculate relative mRNA expression normalized on the reference gene and the corresponding control (undifferentiated or untreated cells) [31]. The RT-qPCR primer pairs used are listed in Table 1. The source of the *Col1α2* primer pair is OriGene Technologies GmbH (Herford, Germany).

### 2.11. Western Blot

For the determination of expressed protein, the hFOB 1.19 cells were seeded at 100% confluency and incubated with cytokines for 2 or 21 days in OIM. For total protein extraction, cells were harvested by scraping and lysed in RIPA buffer supplemented with phosphatase and protease inhibitor cocktail. After sonication (10 pulses, 0.5 s at 80% amplitude), samples were centrifuged at maximum speed for 1 min at 4 °C. The supernatant was collected and stored at −80 °C until use. Protein concentration was determined using the BCA assay (Thermo Fisher Scientific GmbH, Karlsruhe, Germany) according to the manufacturer’s protocol.

Then, 25–30 µg of protein was diluted in 4× Laemmli buffer containing β-mercaptoethanol, heated at 95 °C for 5 min, and loaded onto 4–15% gradient stain-free SDS-polyacrylamide gels. After gel imaging for total protein normalization, proteins were transferred onto PVDF membranes using wet transfer (25 V, 2.5 A, 10 min). Membranes were blocked for 1 h in 5% bovine serum albumin (BSA) in TBS-T (20 mM Tris-HCL, 150 mM NaCl, 0.1% Tween-20), followed by incubation with primary antibodies for overnight at 4 °C. The used primary antibodies and dilutions are listed in Table 2. After washing with TBS-T, membranes were incubated with HRP-conjugated secondary antibodies anti-rabbit or anti-mouse (1:8000 in TBS-T) for 1 h. Signals were detected using an enhanced chemiluminescence (ECL) substrate (Thermo Fisher Scientific GmbH, Karlsruhe, Germany) and visualized on a ChemiDoc^™^ MP Imaging system (Bio-Rad Laboratories, Hercules, CA, USA). Quantification of protein bands was performed using Image Lab software version 6.0.1 (Bio-Rad), and target protein levels were normalized to total protein.

### 2.12. Statistics

The statistical analyses were performed using the GraphPad PRISM 8 software (Boston, MA, USA) or R (Version 4.5.0) with the use of RStudio (Version 2024.9.1.394) (Posit PBC, Boston, MA, USA). The data with hFOB 1.19 cells were treated as parametric, so one-way or two-way ANOVA was used for statistical analysis. *p*-values lower than 0.05 were considered statistically significant.

## 3. Results

### 3.1. Morphological Comparison of hFOB 1.19 Cells and Primary Human Osteoblasts

**Figure 1:**
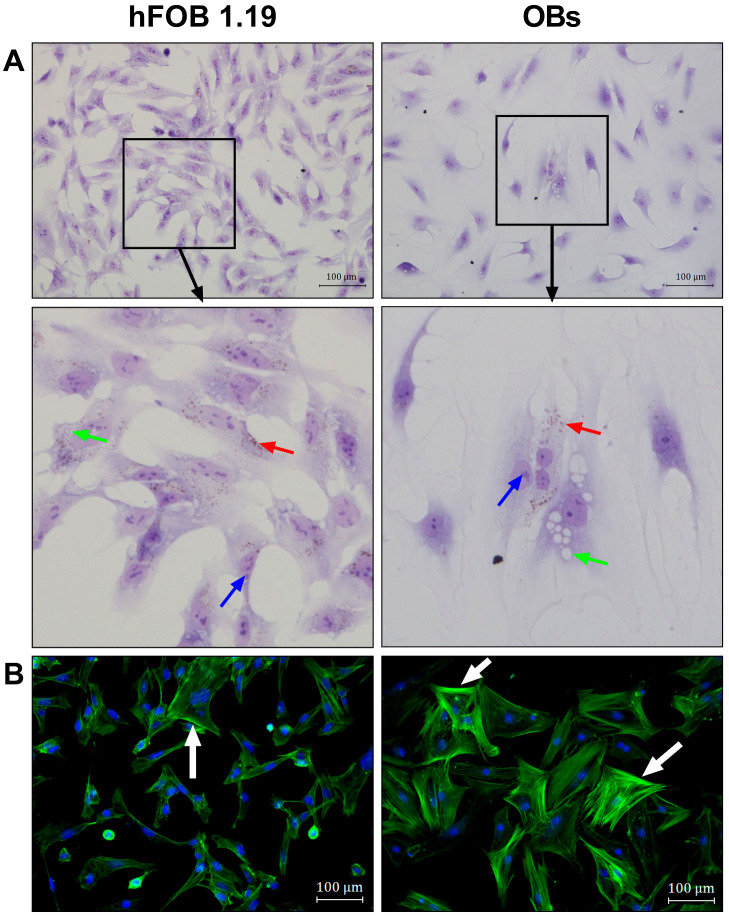
Morphological comparison of hFOB 1.19 cells and primary human osteoblasts (OBs). (**A**) Representative bright-field microscopy images of hFOB 1.19 cells and primary OBs stained with toluidine blue and oil red O. Magnified sections illustrate lipid droplets (red arrows), heterochromatin in cell nuclei (blue arrows), and cytoplasmic vacuole-like structures (green arrows). (**B**) Fluorescence microscopy images showing cytoskeletal organization (F-actin labeled by phalloidin, green) and nuclear DNA (DAPI, blue) with arrows indicating areas of condensed actin cytoskeleton. All microscopy images were taken at 200× magnification (scale bar = 100 µm).

To compare the morphological characteristics of hFOB 1.19 cells and primary human osteoblasts (OBs), toluidine blue, oil red O, and phalloidin stainings were performed (Figure 1). In bright-field images, hFOB 1.19 cells appeared to be smaller (with a size of about 100 µm) and more elongated than OBs (size > 100 µm), with both cell types exhibiting predominantly spindle-shaped morphologies (Figure 1A). In contrast to hFOB 1.19 cells, primary OBs showed a more heterogeneous morphology, including both spindle-shaped and polygonal cells of variable sizes. Additional notable morphological features include lipid droplets (red arrows), which were present in both cell types but predominantly observed in hFOB 1.19 cells, whereas only a small number of cells in the OB cultures contained them. Moreover, large vacuole-like structures were observed in the cytoplasm of some OB cells (green arrows); similar structures were also present in hFOB 1.19 cells but appeared smaller in size. Notably, the nuclei of hFOB 1.19 cells exhibited a more pronounced accumulation of heterochromatin compared to the OB nuclei (blue arrows).

The fluorescence imaging of the actin cytoskeleton revealed differences in the cytoskeletal organization between the cell line and primary cells (Figure 1B). Both the hFOB 1.19 and OBs formed long and pronounced actin stress fibers; however, the OB cultures showed more cells with thick fibers localized along the cell margins than hFOB 1.19 cells.

### 3.2. A Comparison of the Osteogenesis of hFOB 1.19 Cells and Primary Human Osteoblasts

The osteogenic differentiation potential of hFOB 1.19 cells and OBs was compared by assessing extracellular matrix mineralization and the mRNA expression of key osteogenic differentiation markers over 35 days in an osteogenic induction medium (OIM).

**Figure 2:**
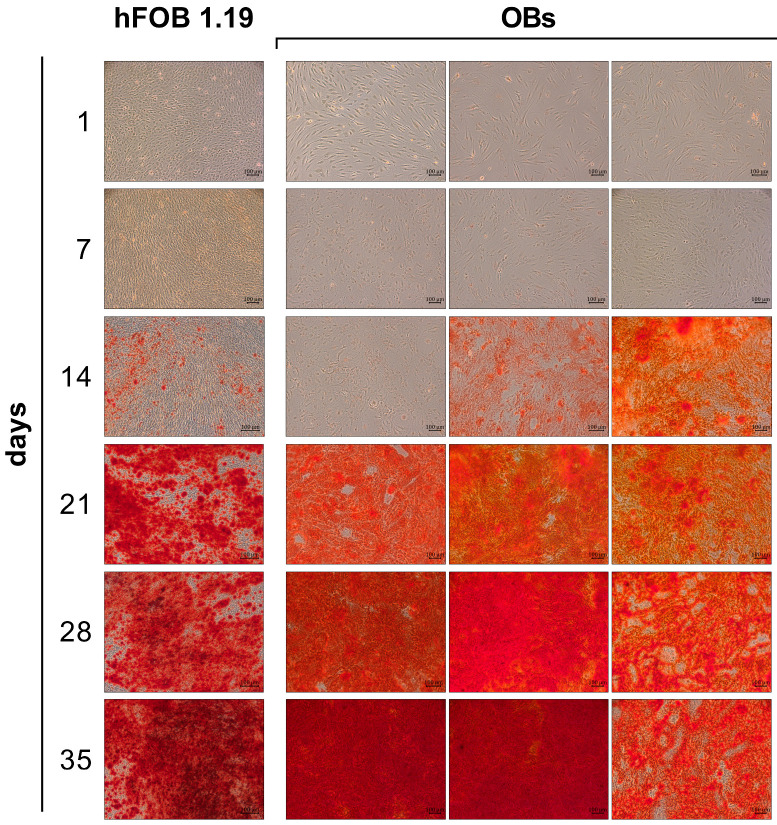
Comparison of matrix mineralization during osteogenic differentiation of hFOB 1.19 cells and primary OBs. Representative bright-field microscopy images of extracellular matrix mineralization visualized by alizarin red S staining at indicated days (1, 7, 14, 21, 28, and 35) of osteogenic induction by osteogenesis induction medium (OIM) for hFOB 1.19 cells and OBs from three different donors. Magnification: 100×, scale bar = 100 µm.

Extracellular matrix mineralization was visualized using alizarin red S staining on days 1, 7, 14, 21, 28, and 35 of osteogenesis (Figure 2). In hFOB 1.19 cells, no mineral deposition was observed at early time points (day 1 and 7). Initial calcium deposits became detectable at day 14 as isolated red-stained nodules. With the increasing culture time, the mineralized areas expanded; yet, even by day 35, the staining did not cover the entire monolayer, leaving unstained regions within the cell lawn.

In contrast, OB cultures from three donors exhibited donor-specific differences in the onset, pattern, and extent of matrix mineralization. In two donors, the initial calcium deposit became visible between day 7 and 14, similar to hFOB 1.19 cells, while, in the third donor, mineral deposition began between day 14 and 21. Despite the delayed onset, this third donor showed a rapid progression thereafter, reaching dense and confluent mineralization by day 35. One of the early-mineralizing donors also achieved full surface coverage by day 35, whereas the other showed a distinct mineralization pattern characterized by a less confluent calcium deposition.

The expression profiles of key osteogenic differentiation markers—*alkaline phosphatase* (*Alp*), *collagen type I alpha 2* (*Col1α2*), *runt-related transcription factor 2* (*Runx2*), *osteopontin* (*Opn*), and *osteocalcin* (*Ocn*)—were analyzed in hFOB 1.19 cells and primary OBs during a 35-day incubation in OIM.

**Figure 3:**
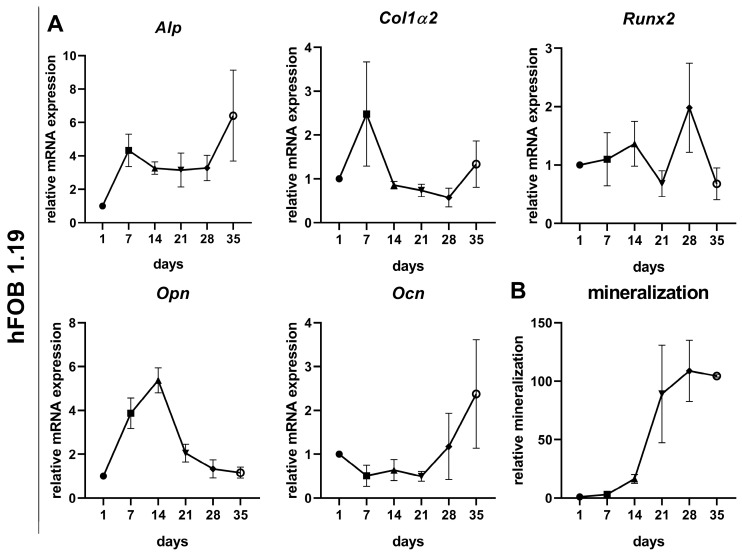
Temporal expression of osteogenic differentiation markers in hFOB 1.19 cells during induced osteogenesis. (**A**) Relative mRNA expression levels of *alkaline phosphatase* (*Alp*), *collagen type 1 alpha 2* (*Col1α2*), *runt-related transcription factor 2* (*Runx2*), *osteopontin* (*Opn*), and *osteocalcin* (*Ocn*) were quantified by qPCR from day 1 to 35 in OIM-treated hFOB 1.19 cells (*n* = 3). Data (mean ± SD) were normalized using the 2^−ΔΔCt^ method relative to day 1 control cells. (**B**) Relative matrix mineralization assessed by alizarin red S staining on days 1, 7, 14, 21, 28, and 35 of osteogenic induction (mean ± SD, *n* = 3).

In hFOB 1.19 cells, *Alp* mRNA levels showed a rapid initial increase between day 1 and 7, followed by a stable expression phase up to day 28 (Figure 3A). At day 35, *Alp* reached its maximum. The *Col1α2* expression peaked at day 7 and declined markedly thereafter. The *Runx2* expression fluctuated over time, with a slight increase from day 1 to 14, a drop at day 21, a transient peak at day 28, and a decrease by day 35. The *Opn* expression increased early, reaching its maximum at day 14, followed by a progressive decline through day 35. In contrast, *Ocn* mRNA levels remained low during the early phase of differentiation, but started to increase from day 21 onward, culminating in a marked upregulation at day 35. The quantitative analysis of matrix mineralization revealed the strongest increase between days 14 and 21, followed by a plateau from day 28 onwards (Figure 3B).

**Figure 4:**
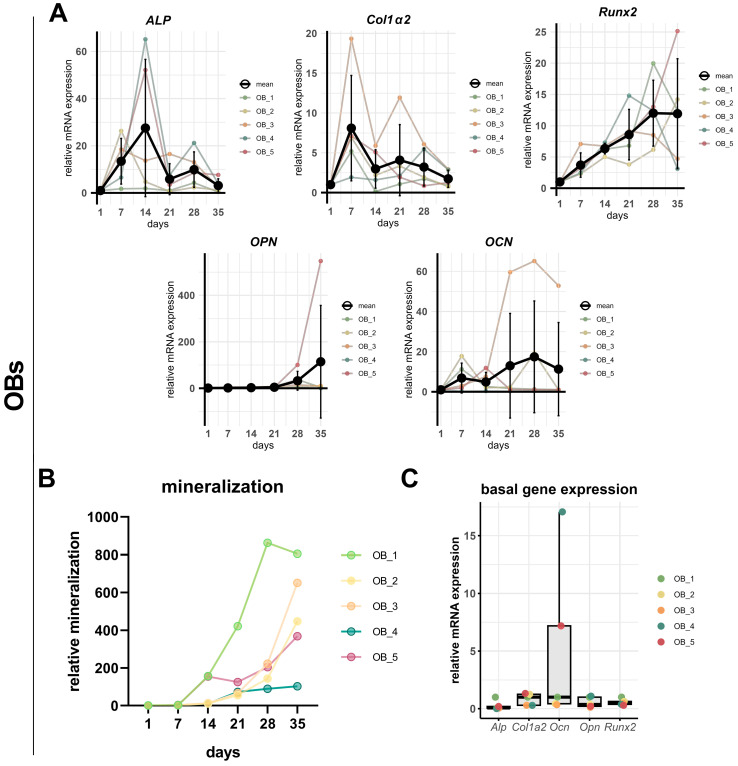
Individual variations in osteogenic differentiation marker expression in primary OB cultures during induced osteogenesis. (**A**) Relative mRNA expression levels of *Alp*, *Col1α2*, *Runx2*, *Opn*, and *Ocn* from day 1 to 35 of OIM treatment in OBs derived from five individual donors, showing individual data points and mean ± SD (*n* = 5), normalized to day 1 expression for each donor by the 2^−ΔΔCt^ method. (**B**) Individual relative mineralization profiles assessed by alizarin red S staining on indicated days (1, 7, 14, 21, 28, and 35) of OIM treatment for each donor. (**C**) Basal (undifferentiated) gene expression of *Alp*, *Col1α2*, *Runx2*, *Opn*, and *Ocn*, normalized relative to OB_1 by using the 2^−ΔΔCt^ method. Individual values and mean ± SD are shown.

In primary OBs, gene expression patterns were significantly variable across donors but showed overall stronger induction compared to hFOB 1.19 cells. In Figure 4A, mean expression levels are shown alongside individual donor expression dynamics to illustrate the inter-individual variability. The *Alp* expression increased between day 1 and 14, followed by a decline toward day 35. However, the donor-specific profiles showed variation in both peak timing and expression amplitude, with one donor (OB_3) maintaining high *Alp* levels until day 28. *Col1α2* expression peaked consistently at day 7 across donors, with the exception of donor OB_4. The *Opn* expression increased over time, reaching peak levels between day 28 and 35 of all donors. However, the magnitude of induction varied markedly, with one donor (OB_5) having a disproportionately strong upregulation that exceeded the levels of all others. This donor-specific outlier contributed substantially to the overall variance observed in the group. The expression of *Ocn* also resulted in substantial inter-donor variability. OB_3 cells exhibited a strong and sustained increase from day 14 onward, peaking at day 28 and 35 with a high magnitude. Another had a biphasic pattern with early and late peaks, while two donors’ *Ocn* expression peaked in the earlier differentiation phase (day 7 or 14). No clear pattern emerges when the expression of these osteogenic markers is assessed separately for each donor (see Supplementary Figure S1). The *Runx2* expression exhibited a uniform trend, with all donors progressively increasing from day 1, with three donors reaching its maximum before day 35. *Runx2* was the only marker that was proportional to the increasing mineralization (Figure 4B).

As the expression levels in the time course analyses are normalized to day 1 for each donor, Figure 4C shows the basal expression levels of the individual donor cells in their undifferentiated state. The expression levels of *Alp*, *Col1α2*, *Runx2*, and *Opn* were comparably expressed across donors, whereas the *Ocn* expression showed pronounced inter-donor variability, with two donors (OB_4 and OB_5) exhibiting substantially elevated baseline expression levels.

### 3.3. Effects of Proinflammatory Cytokines on the Osteogenesis and Proliferation of hFOB 1.19 Cells

Having compared the osteogenic differentiation potential of hFOB 1.19 cells and OBs, we next examined the responsiveness of hFOB 1.19 cells to inflammatory stimuli. In a recently published study, we found that proinflammatory cytokines, particularly interleukin-1 beta (IL-1β) and tumor necrosis factor alpha (TNF-α), enhanced matrix mineralization in primary OBs [8]. To assess whether hFOB 1.19 cells reflect similar behavior, we analyzed the effect of IL-1β, IL-6, and TNF-α on matrix mineralization and ALP activity in this cell line.

**Figure 5:**
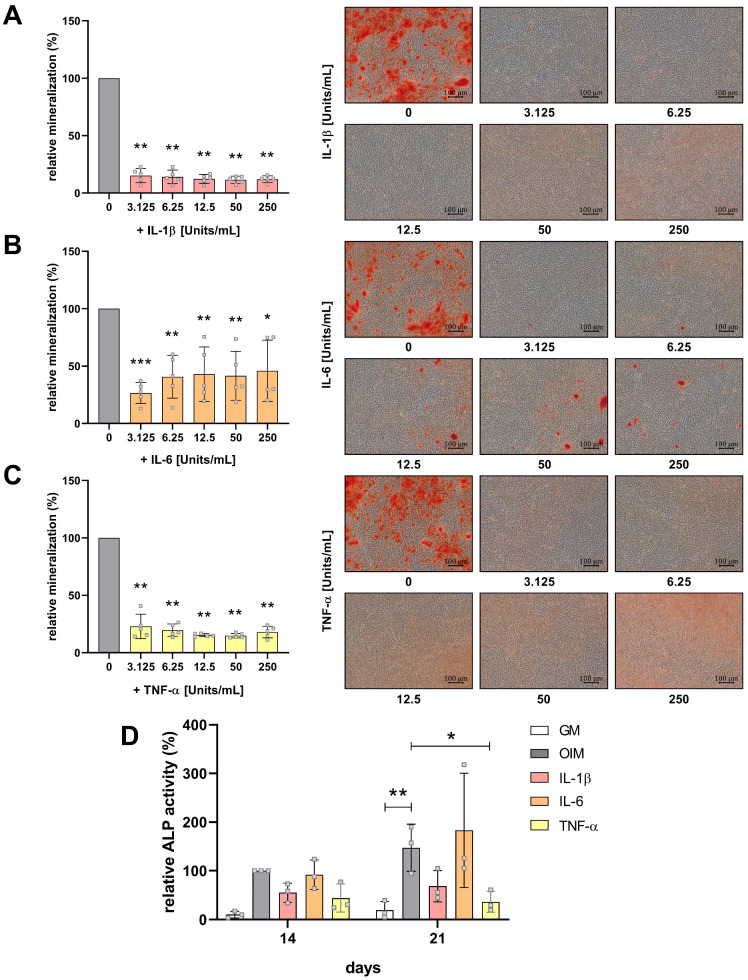
Proinflammatory cytokines inhibit the osteogenesis of hFOB 1.19 cells. Matrix mineralization of hFOB 1.19 cells incubated with (**A**) interleukin (IL)-1β, (**B**) IL-6, and (**C**) tumor necrosis factor alpha (TNF-α) at concentrations ranging from 3.125 to 250 Units/mL in OIM was quantified using alizarin red S staining at day 21 (*n* = 5). Representative microscopic images (100× magnification, scale bar = 100 µm) are shown. (**D**) Relative alkaline phosphatase (ALP) activity of hFOB 1.19 cells incubated in growth medium (GM), OIM, and OIM with cytokines (IL-1β, 6.25 Units/mL; IL-6, 12.5 Units/mL; TNF-α, 6.25 Units/mL) on days 14 and 21 (*n* = 3), normalized to cell number quantified by MTT assays. Statistical analysis was performed using one-way ANOVA (panels **A**–**C**) and two-way ANOVA (panel **D**), followed by Dunnett’s multiple-comparisons test (*p* ≤ 0.05 (*), *p* ≤ 0.01 (**), *p* ≤ 0.001 (***)). Unconnected asterisks denote comparisons to cytokine-free differentiated control (0 Units/mL). Data are presented as mean ± SD.

hFOB 1.19 cells were cultured in OIM for 21 days with and without cytokines at concentrations ranging from 3.125 to 250 U/mL. Alizarin red S staining revealed a strong inhibitory effect of all three cytokines on matrix mineralization, which was already statistically significant at the lowest concentrations tested (Figure 5A–C). IL-1β and TNF-α completely inhibited the formation of mineralized nodules. At higher doses—particularly with TNF-α—a faint reddish background was observed microscopically. However, this did not result in measurable mineral accumulation, as confirmed by quantitative analysis.

IL-6 treatment also inhibited the matrix mineralization, but small mineralized nodules remained visible at all concentrations, indicating the partial preservation of mineralization. However, these nodules were fewer in number and appeared larger and more isolated compared to the finely distributed nodules seen in OIM-stimulated controls at day 14 (Figure 2). Interestingly, the strongest inhibitory effect of IL-6 was observed at the lowest concentration (3.125 U/mL).

In addition to matrix mineralization, ALP activity was measured on days 14 and 21 (Figure 5D). With OIM treatment, ALP activity increased over time and was significantly higher than untreated cells in the growth medium (GM). In contrast to IL-6, IL-1β and TNF-α significantly inhibited the ALP activity at both time points compared to OIM-treated cells.

To examine the impact of the proinflammatory cytokines on osteogenic marker expression, the relative mRNA levels of *Alp*, *Col1α2*, *Runx2*, and *Opn* were analyzed in hFOB 1.19 cells after 21 days of differentiation in OIM with or without cytokine treatment.

**Figure 6:**
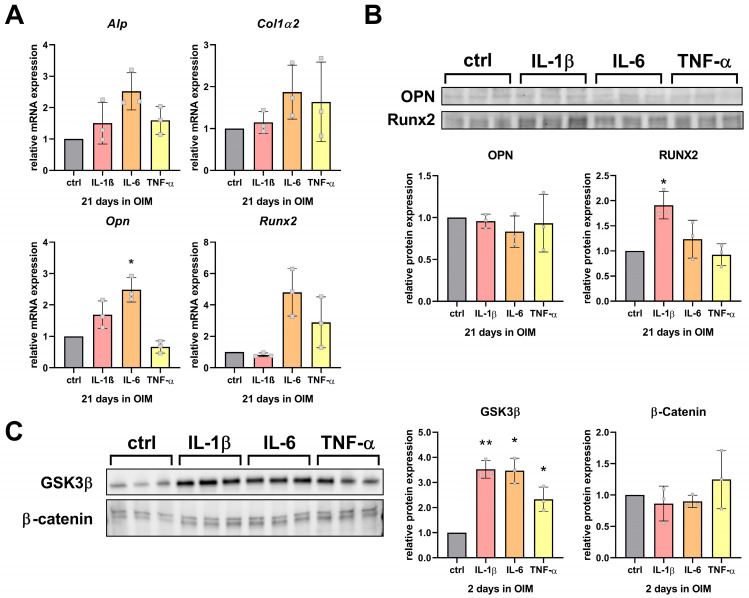
Cytokine treatment modulates osteogenic marker expression in hFOB 1.19 cells. (**A**) mRNA expression levels of osteogenic differentiation markers (*Alp*, *Col1α2*, *Runx2*, and *Opn)* measured via qPCR after 21 days of treatment in OIM supplemented with cytokines (IL-1β, 6.25 Units/mL; IL-6, 12.5 Units/mL; TNF-α, 6.25 Units/mL). Gene expression data were analyzed by the 2^(−ΔΔCt)^ method and normalized to the cytokine-free control cells (ctrl; OIM only). (**B**) Protein levels of osteogenic markers OPN and RUNX2 determined by Western Blot at day 21 (30 µg total protein loaded). (**C**) Protein expression of signaling proteins Glycogen synthase kinase-3 β (GSK3β) and β-catenin after 2 days of cytokine treatment assessed by Western blot (25 µg total protein loaded). Protein bands were normalized to total protein. Data are shown as mean ± SD (*n* = 3). Statistical analysis was performed by one-way ANOVA followed by Dunnett’s multiple-comparisons test (*p* ≤ 0.05 (*), *p* ≤ 0.01 (**)).

IL-6 significantly increased the mRNA expression of *Opn*, and also showed a trend to upregulate *Alp*, *Runx2*, and *Col1α2* expression (Figure 6A). In contrast, IL-1β and TNF-α did not induce significant changes in osteogenic marker expression, although the *Runx2* and *Col1α2* expression were tendentially upregulated and *Opn* expression was downregulated with TNF-α treatment compared to IL-1β treatment. At the protein level, the effects of IL-6 on OPN and RUNX2 could not be confirmed. None of the cytokines altered the OPN protein levels on day 21, whereas IL-1β significantly increased the RUNX2 protein expression (Figure 6B).

Interestingly, at an early stage of osteogenic differentiation (day 2 in OIM), treatment with all cytokines resulted in a significant increase in Glycogen synthase kinase-3 β (GSK3β) protein expression, while β-catenin protein levels remained unchanged at this time point (Figure 6C).

Since all three cytokines significantly inhibited the matrix mineralization of hFOB 1.19 cells, we next examined whether these effects were associated with changes in cell proliferation.

**Figure 7:**
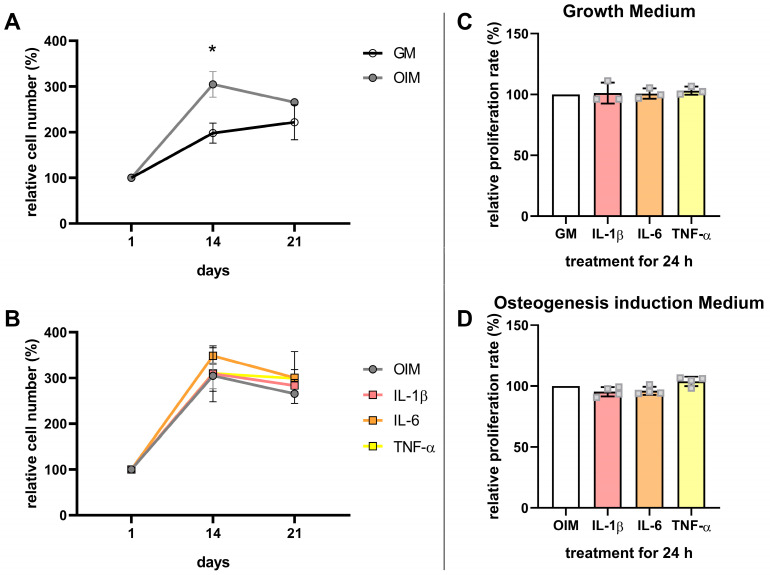
Proinflammatory cytokines do not affect the proliferation of hFOB 1.19 cells. (**A**,**B**) Relative cell number quantified via MTT assay at days 1, 14, and 21 in GM and OIM (**A**) or in OIM with and without cytokines (IL-1β, 6.25 Units/mL; IL-6, 12.5 Units/mL; TNF-α, 6.25 Units/mL) (**B**), normalized to day 1 (*n* = 3). (**C**,**D**) Relative proliferation rate measured by BrdU incorporation assay in GM (**C**, *n* = 3) or OIM (**D**, *n* = 4), following 24 h of cytokine treatment. Data were normalized to the untreated control (GM or OIM). Data are shown as mean ± SD. Statistical significance was determined by one-way ANOVA followed by Dunnett’s multiple-comparisons test (*p* ≤ 0.05 (*)).

Cell numbers were analyzed by MTT assays on days 1, 14, and 21. In the absence of cytokines, cells cultured in OIM showed significantly higher cell numbers than those in GM at day 14, while values at day 21 were comparable between both conditions (Figure 7A). Across all cytokine-treated groups, cell numbers on days 14 and 21 were comparable to the OIM control (Figure 7B). Consistent with these findings, IL-1β, IL-6, and TNF-α also did not affect the proliferation rates of the cells incubated in GM or OIM, measured by BrdU incorporation after 24 h (Figure 7C,D).

## 4. Discussion

In the present study, we demonstrated that the proinflammatory cytokines IL-1β, IL-6, and TNF-α significantly inhibit the mineralization of the hFOB 1.19 cell line, with IL-1β and TNF-α having the strongest impact with a significant inhibition of ALP activity. Cytokine treatment did not affect the cell proliferation nor inhibited the expression of osteogenic differentiation markers. The response of hFOB 1.19 cells to proinflammatory cytokines is consistent with previous publications using in vitro cell models such as MC3T3-E1 or murine MSCs [3,32]. On the other hand, this outcome stands in direct contrast to our recently published data in which these cytokines significantly promoted the mineralization of primary OBs [8]. This contrasting response suggests that there are fundamental differences between the two models.

A possible explanation for the opposing effects could lie in the differences in their differentiation stages. While primary OB cultures had the characteristics of an advanced osteogenic stage, the hFOB 1.19 cell line appears to correspond to a significantly earlier stage of osteogenic differentiation. The primary OB cultures showed a heterogeneous cell pattern with both spindle and polygonal-shaped cells, indicating a mixed population of various differentiation stages of osteoblasts. In contrast, hFOB 1.19 cells were smaller, predominantly spindle-shaped, and showed a less pronounced actin cytoskeleton with weaker stress fibers. These features suggest an earlier, pre-osteoblast or even mesenchymal stem cell stage [33]. Stronger stress fibers in the peripheral cell region and polygonal shape in the OBs correlate with a more advanced osteogenic phenotype [34,35]. In addition, hFOB 1.19 cells showed increased heterochromatin in the nucleus—an indication of lower transcriptional activity and a more quiescent cell state compared to the OBs [36]. There is further evidence in the literature of the immature state of hFOB 1.19 cells, such as the expression of the MSC markers CD73 and CD105, as well as their ability to differentiate into adipogenic cells [37]. hFOB 1.19 cells thus tend to exhibit characteristics of very early pre-osteoblasts or MSCs, in which cytokines have been shown to inhibit their osteogenic differentiation [3,32,38,39]. Consequently, more mature osteoblasts could be promoted in their mineralization by cytokines, as we have shown with primary OBs. This hypothesis is further supported by a study that demonstrated that MSCs that were osteogenically pre-differentiated exhibited significantly increased mineralization in response to TNF-α [15]. Another study using fetal calvarial progenitor cells showed a differentiation-dependent response to TNF-α, wherein the differentiation of undifferentiated cells was inhibited, while TNF-α did not affect pre-differentiated cells [18]. This observation could also have great clinical relevance. The pre-differentiation of MSCs or osteogenic progenitor cells before the exposure to inflammatory stimuli may represent a promising therapeutic strategy. It also has implications for regenerative therapies, where the use of pre-differentiated cells may help avoid the cytokine-induced inhibition of bone formation. The modulation of inflammation could hold great potential, but also risks, as prolonged cytokine treatment could, in turn, lead to bone destruction, as can be seen in chronic inflammatory diseases such as rheumatoid arthritis [40,41]. To translate the potential of differentiation-dependent cytokine responses into clinical applications, further research is needed. Specifically, it could be essential to identify the ‘cut-off point’ which defines the differentiation threshold at MSCs shift from a stage where cytokines like IL-1β, TNF-α, or IL-6 inhibit osteogenesis to one where cytokines may promote mineralization.

Several studies have shown that proinflammatory cytokines can promote the mineralization of hMSCs, comparable to our previous results with human OBs [5,6]. The analysis of potential signaling pathways responsible for the different cytokine responses of cell models and primary human cells, such as Wnt/β-catenin, MAPK, or NF-κB, could be of great interest [42]. It was shown that the TNF-α treatment of murine MSCs led to enhanced β-catenin degradation via the activation of the NF-κB signaling pathway, while the activation of NF-κB by TNF-α in human MSCs promoted osteogenic differentiation [39,43]. The observed upregulation of GSK3β protein expression in our study, a known negative regulator of the Wnt/β-catenin signaling pathway, supports a cytokine-mediated inhibition of this pathway as seen in murine MSCs. However, the β-catenin protein levels of hFOB 1.19 cells remained unchanged. An analysis of the NF-κB and MAPK pathways revealed a trend toward ERK1/2 (p42/p44 MAPK) activation especially by IL-1β, while phosphorylated NF-κB was undetectable, suggesting a limited activation of the NF-κB pathway (Figure S2). Interestingly, inhibiting the ERK1/2 pathway is a promising therapeutic target in patients with rheumatoid arthritis [44]. In MC3T3-E1 cells, IL-6 has been shown to influence osteogenic differentiation through various signaling pathways, with the activation of the SHP2/ERK axis resulting in the inhibition of osteogenesis [38]. Follow-up studies investigating the dynamic signaling responses to cytokines over time will be necessary in order to clarify these findings. The complete inhibition of calcium nodule formation by IL-1β and TNF-α may also result from impaired matrix formation, potentially mediated by the induction of matrix metalloproteinases [45]. To test whether excessive matrix degradation contributes to the inhibition of mineralization, we evaluated the protein expression of MMP2 and the inhibitors TIMP2 and TIMP3 on day 21 in hFOB 1.19 cells under cytokine treatment (Figure S3). Unexpectedly, the MMP2/TIMP2 ratio was significantly lower in TNF-α-treated cells than in untreated controls, suggesting that the observed inhibition of matrix mineralization is less likely due to the enhanced matrix degradation by MMPs. Instead, it may be attributed to alternative mechanisms or impaired extracellular matrix formation.

The observed inhibition of mineralization in the absence of ALP suppression under IL-6 treatment suggests the involvement of ALP-independent regulatory pathways. A plausible mechanism may involve the significantly increased mRNA expression of *Opn* by IL-6, as OPN is known to bind hydroxyapatite and act as a mineralization inhibitor by preventing crystal growth within the extracellular matrix [46]. In contrast, ALP dephosphorylates OPN, thereby removing its inhibitory activity and preventing OPN from binding calcium [47]. Since ALP activity is inhibited by IL-1β and TNF-α in hFOB 1.19 cells, this could explain the stronger inhibition of mineralization compared to IL-6. Together with the trend of increased *Alp* and *Col1α2* expression under IL-6, this suggests proper matrix formation but impaired matrix mineralization. Analyzing the matrix composition, in particular, the levels of secreted phosphorylated OPN, could help determine whether cytokines specifically inhibit mineralization while osteogenic differentiation itself remains unaffected in hFOB 1.19 cells. The involvement of this mechanism remains speculative, as OPN protein levels were neither elevated nor reduced by cytokine treatment. The up- and downregulation of osteogenic markers at specific time points is often interpreted as a sign of increased or decreased osteogenesis (e.g., [14]). When the temporal expression patterns were considered, the upregulation of *Opn* on day 21 may indicate a differentiation stage more similar to early osteogenesis (about day 7), rather than reflecting a progression toward terminal maturation. This interpretation aligns with the reduced mineralization and suggests that cytokine treatment may interfere with the transition from early differentiation to functional matrix mineralization. Alternatively, the increased expression of osteogenic marker genes could also represent a compensatory feedback mechanism in response to impaired mineralization.

Despite the possible different maturation stages, both cell types showed a comparable onset of mineralization between days 14 and 21 of osteogenic induction. In the OB cultures, however, a variance between donors was visible, both concerning the onset of calcium phosphate formation and in the overall increase up to day 35. While mineralization in hFOB 1.19 cells increased by a factor of about 100, the mineralization of OBs increased by 100 to 800 times, depending on the donor. The analysis of the osteogenic marker profiles in hFOB 1.19 cells largely showed the typical expression pattern for the differentiation stages of osteogenesis. In the phase of extracellular matrix maturation, *Alp* and *Col1α2* were upregulated from day 7, together with *Opn*, which is considered an intermediate or late differentiation marker [48]. From day 14, these expressions decreased significantly, indicating a phase change to the mineralization phase. When the development of mineralization was already stagnating, the *Ocn* expression was increased from day 28 onwards.

In primary OBs, the temporal pattern of the osteogenic marker expression showed considerable inter-individual variability. A key observation was the high variability in *Ocn* expression, both in timing and intensity. Interestingly, among undifferentiated cells, *Ocn* was the only marker showing a strong donor-specific basal expression. Notably, the donor with the highest basal *Ocn* expression (OB_4) exhibited the lowest mineralization rate. To clarify whether a direct correlation exists, future studies with a greater number of donor cell samples are necessary. The expression patterns of other markers such as *Alp* and *Opn* were more consistent across donors: Comparable to hFOB 1.19 cells, *Alp* was upregulated early (days 7–14) in all OBs and *Col1α2* peaked around day 7, except in one donor with a delayed increase at day 28. *Opn* was expressed at later stages (days 21–35). These donor differences may be attributed to genetic or epigenetic factors, culture heterogeneity, or other unknown variables. Notably, the mRNA expression of *Runx2* was correlated with the increasing mineralization over time in all donors. Overall, the data show the limitations of interpreting the osteogenic marker expression at specific time points, in cell lines as well as in primary cell cultures due to variability, without assessing the dynamic marker expression over time. The conclusion that it is not sufficient to select single days as a standard for expression analysis was already drawn in a study directly comparing the osteoblast cell lines MG-63, MC3T3-E1, and SaOS-2 cells to primary OBs [49]. In our study, *Runx2* was the only marker that consistently correlated with the increase in mineralization in OBs, but this cannot be confirmed with the hFOB 1.19 cell line. *Runx2* is required for the initiation of osteogenesis and remains expressed in pre- and immature osteoblasts. Its upregulation does not necessarily reflect the maturation, in contrast to other markers such as *Ocn* and *Opn,* whose expression is regulated downstream of *Runx2* [50]. Interindividual differences in mineralization and osteogenic marker expression among primary OBs warrant further investigation in a larger cohort to explore the potential associations with disease progression or patient comorbidities.

It must be acknowledged that the use of a cell line introduces inherent limitations. Although hFOB 1.19 cells showed comparable osteogenic differentiation in terms of mineralization and expressed typical osteogenic markers, their immortalization and the homogeneity of the culture limit their direct comparability to primary cells like MSCs or preosteoblasts. Another important factor is the fetal origin of the cell line, whereas primary OBs in this study were derived from elderly patients (with an average age of 74 years). A comparison between adult and fetal bone marrow MSCs revealed significant differences in the expression of osteogenic markers [51]. A different expression profile, especially of immunologically relevant genes, may also lead to distinct pathway activations by cytokines in fetal cells. Moreover, the addition of components (F-12 nutrient mix and G418) in the basal media for hFOB 1.19 cells could also contribute to the inter-model differences shown in this study. While both media for OBs and hFOB 1.19 cells support osteogenic differentiation and cells show comparable baseline mineralization under control conditions (Figure 2), subtle effects on cytokine signaling cannot be ruled out. However, the robust and consistent response patterns observed within each model, combined with previous reports showing divergent cytokine effects across cell types cultured in identical base media, support that cytokine responsiveness is predominantly driven by intrinsic cell-type properties [15,52]. Further studies are needed in order to assess the impact of different media components on cytokine and osteogenic signaling. While these differences complicate direct comparisons between hFOB 1.19 cells and primary OBs, they do not compromise the significance of the conclusions drawn within each model system.

## 5. Conclusions

In conclusion, this study demonstrates that proinflammatory cytokines strongly inhibit the osteogenesis of hFOB 1.19 cells, with IL-1β and TNF-α exerting the most pronounced effects. This outcome stands in clear contrast to the findings with primary human OBs, where the same cytokines enhanced mineralization. Such contradictions have been repeatedly observed in the literature, but their underlying mechanisms remain unclear due to the complexity of cytokine and osteogenic signaling. One possible explanation is the differentiation stage of hFOB 1.19 cells and OBs, due to the pre-osteoblastic or mesenchymal-like morphological features of hFOB 1.19 cells, which has more frequently been associated with the cytokine-induced inhibition of osteogenesis, e.g., with the cell line MC3T3-E1. Additional contributing factors may include the homogeneity of the cell line compared to the heterogeneous composition of primary cell populations, as well as the fetal origin of hFOB 1.19. Moreover, while cell lines like hFOB 1.19 reflect the characteristics of a single donor, primary OBs exhibit substantial interindividual variability in both the mineralization capacity and osteogenic marker expression. This variability underscores the importance of including donor diversity in in vitro models to better reflect the clinical heterogeneity of bone remodeling. While hFOB 1.19 cells may not fully reflect the cytokine responsiveness of mature osteoblasts, they could serve as a useful model to help elucidate the discrepancies reported in the literature regarding cytokine effects on osteogenic differentiation. The contrast between osteoblast cell models highlights the importance of critically evaluating the model system when addressing clinically relevant questions in the context of osteoimmunology.

## Figures and Tables

**Table 1:** List of RT-qPCR Primers.

Target Gene	Forward Primer (5′►3′)	Reverse Primer (5′►3′)	Product Length [bp]	Primer Concentration [µM]
*Opn*	CACTCCAGTTGTCCCCACAGTAG	TCTGTAGCATCAGGGTACTGGATGT	120	0.2
*Runx2*	AGTGGACGAGGCAAGAGTTT	TGTCTGTGCCTTCTGGGTTC	125	0.2
*Alp*	GCAGGCAGCTTGACCTCCTC	GCATGGGGGCCAGACCAAAG	121	0.2
*Rplp0*	TTCTCGCTTCCTGGAGGGTGT	CCAGGACTCGTTTGTACCCGT	113	0.4
*Ocn*	CTCCTCGCCCTATTGGCCCT	CTGCTTGGACACAAAGGCTGC	105	0.3
*Tfrc*	TTCAGGTCAAAGACAGCGCTCA	CTATACGCCACATAACCCCCAGG	100	0.4
*Col1α2*	CCTGGTGCTAAAGGAGAAAGAGG	ATCACCACGACTTCCAGCAGGA	135	0.4

**Table 2:** List of primary antibodies for Western Blot.

Target Protein	Company	Reference Number	Dilution
RUNX2	Santa Cruz Biotechnology (Dallas, TX, USA)	Sc-390351	1:100
OPN	Invitrogen (Carlsbad, CA, USA)	MA5-17180	1:500
β-catenin	Abcam (Cambridge, UK)	Ab16051	1:4000
GSK3β	Cell Signaling Technology (Cambridge, UK)	9315	1:1000

## Data Availability

The data underlying this article will be shared upon reasonable request from the corresponding author.

## Supplementary Materials

The following supporting information can be downloaded at: https://www.mdpi.com/article/10.3390/cells14161264/s1, Figure S1: Profiles of mRNA expression of osteogenic markers in OBs derived from individual donors; Figure S2: Analyses of MAPK and NF-κB signaling pathway activation of cytokine-treated hFOB 1.19 cells on day 2; Figure S3: Protein levels of MMP2, TIMP2, and TIMP3 of cytokine-treated hFOB 1.19 cells on day 21; Figure S4: Images of Western blots and corresponding protein gels for OPN, Runx2, GSK3β, and β-catenin protein expression quantification; Table S1: Patient information of the five OB-donors used for mRNA expression profiles; Table S2: Seeding cell numbers of hFOB 1.19 cells and OBs at different confluences.

## Abbreviations

The following abbreviations are used in this manuscript:

IL-1β	Interleukin 1 beta
IL-6	Interleukin 6
TNF-α	Tumor necrosis factor alpha
OBs	Primary human osteoblast-like cells
ALP	Alkaline phosphatase
MSCs	Human mesenchymal stem cells
Col1	Collagen type I
Runx2	Runt-related transcription factor 2
Ocn	Osteocalcin
Opn	Osteopontin
DXA	Dual-energy X-ray absorptiometry
RT-qPCR	Real-time quantitative polymerase chain reaction
SD	Standard deviation
Pen/Strep	Penicillin/streptomycin
FBS	Fetal bovine serum
HEPES	4-(2-hydroxyethyl)-1-piperazineethanesulfonic acid solution
G418	Geneticin
GM	Growth medium
OIM	Osteogenic induction medium
PFA	Paraformaldehyde
RT	Room temperature
PBS	Phosphate-buffered saline
DAPI	4′,6-diamidino-2-phenylindole
ddH_2_O	Double-distilled water
CPC	(1 hexadecyl) pyridinium chloride monohydrate
MTT	Dimethylthiazolyl Blue Tetrazolium Bromide
DMSO	Dimethyl sulfoxide
BrdU	Bromodeoxyuridine
bp	Base pairs
Rplp0	Ribosomal protein lateral stalk subunit P0
Tfrc	Transferrin receptor protein 1
mRNA	Messenger ribonucleic acid
U	Units
MAPK	Mitogen-activated protein kinase
NF-κB	Nuclear factor ‘kappa-light-chain-enhancer’ of activated B-cells
GSK3β	Glycogen synthase kinase-3 beta
