# Supplementary material for: Comparison of Primary Human Osteoblast-like Cells and hFOB 1.19 Cells: Contrasting Effects of Proinflammatory Cytokines
Source: Cells. 2025 Aug 15;14(16):1264. doi: 10.3390/cells14161264 (PMC12384873; doi:10.3390/cells14161264)

Supplements

List of Figures:

Figure S1: Profiles of mRNA expression of osteogenic markers in OBs derived from individual donors.

Figure S2: Analyses of MAPK and NFκB signaling pathway activation of cytokine-treated hFOB 1.19 cells on day 2.

Figure S3: Protein levels of MMP2, TIMP2, and TIMP3 of cytokine-treated hFOB 1.19 cells on day 21.

Figure S4: Images of Western blots and corresponding protein gels for OPN, Runx2, GSK3β, and β-catenin protein expression quantification.

List of Tables:

Table S1: Patient information of the five OB-donors used for mRNA expression profiles.

Table S2: Seeding cell numbers of hFOB 1.19 cells and OBs at different confluences.

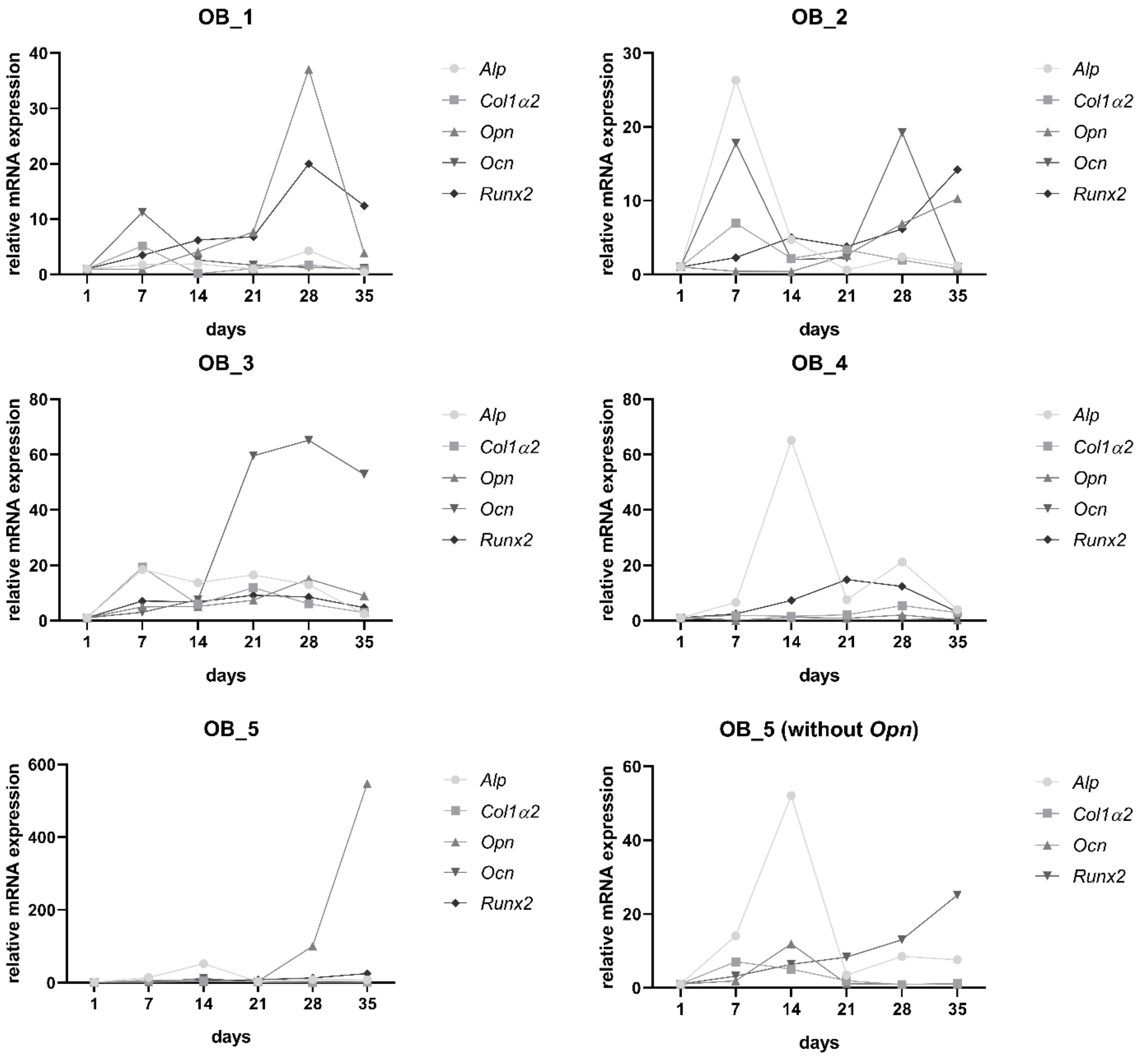

Figure S1: Profiles of mRNA expression of osteogenic markers in OBs derived from individual donors, each graph represents a single donor from the data shown in Figure 4.

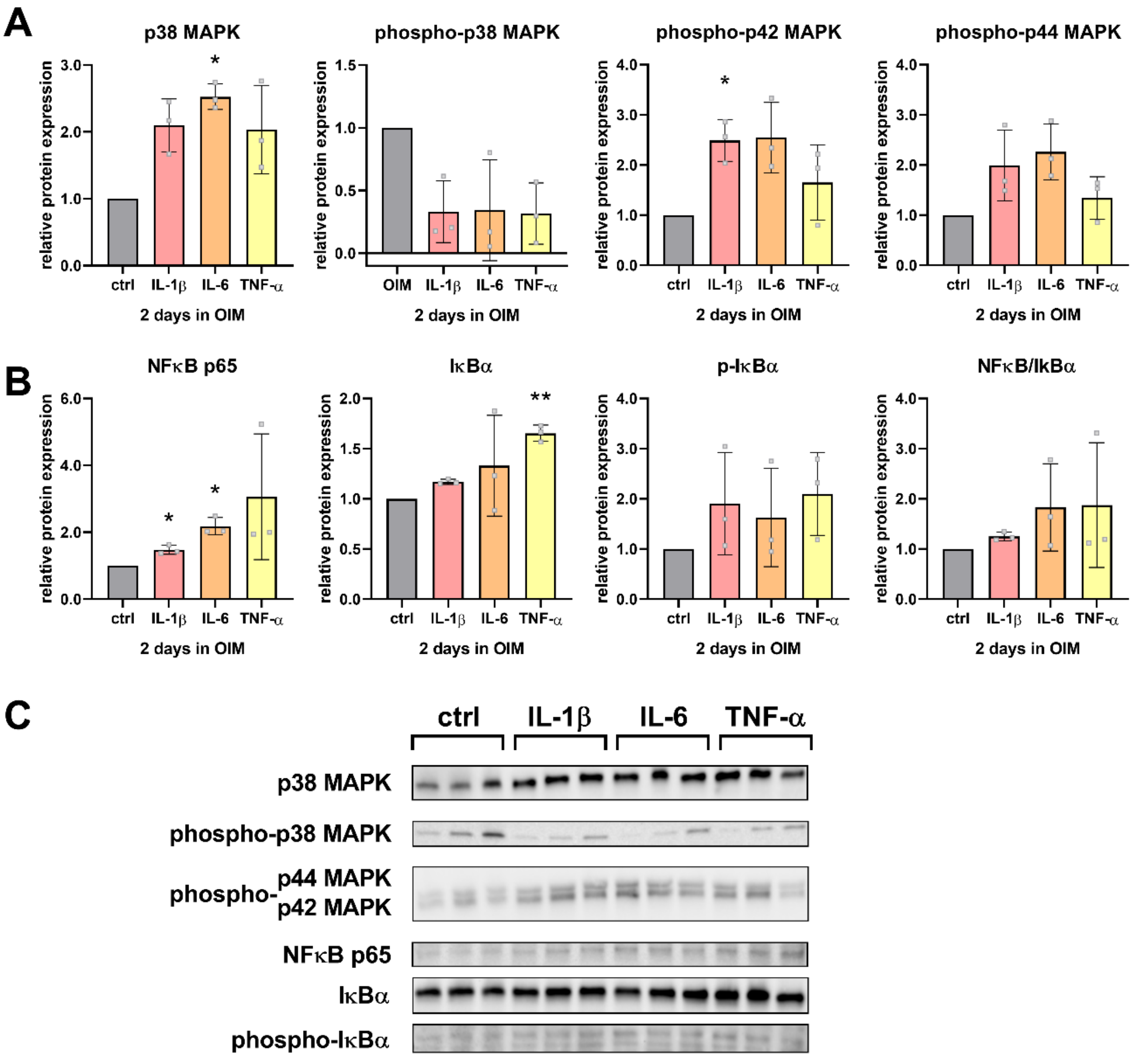

Figure S2: Analyses of MAPK and NFκB signaling pathway activation of cytokine-treated hFOB 1.19 cells on day 2. (A) Protein levels of MAPK signaling pathway components (p38 MAPK, phospho-p38 MAPK, phospho-p42 MAPK, and phospho-p44 MAPK) and of (B) NFκB signaling pathway components (NFκB p65, IκB*α*, phospho-IκB*α*, and the NFκB/IκB*α* ratio) were assessed by Western blotting at day 2 of osteogenesis with cytokine treatment. Protein signals were normalized to total protein (25 µg total protein loaded). Data are shown as mean ± SD (n = 3). Statistical analysis was performed by one-way ANOVA followed by Dunnett's multiple comparisons test ($p \leq 0.05$ (*), $p \leq 0.01$ (**)).

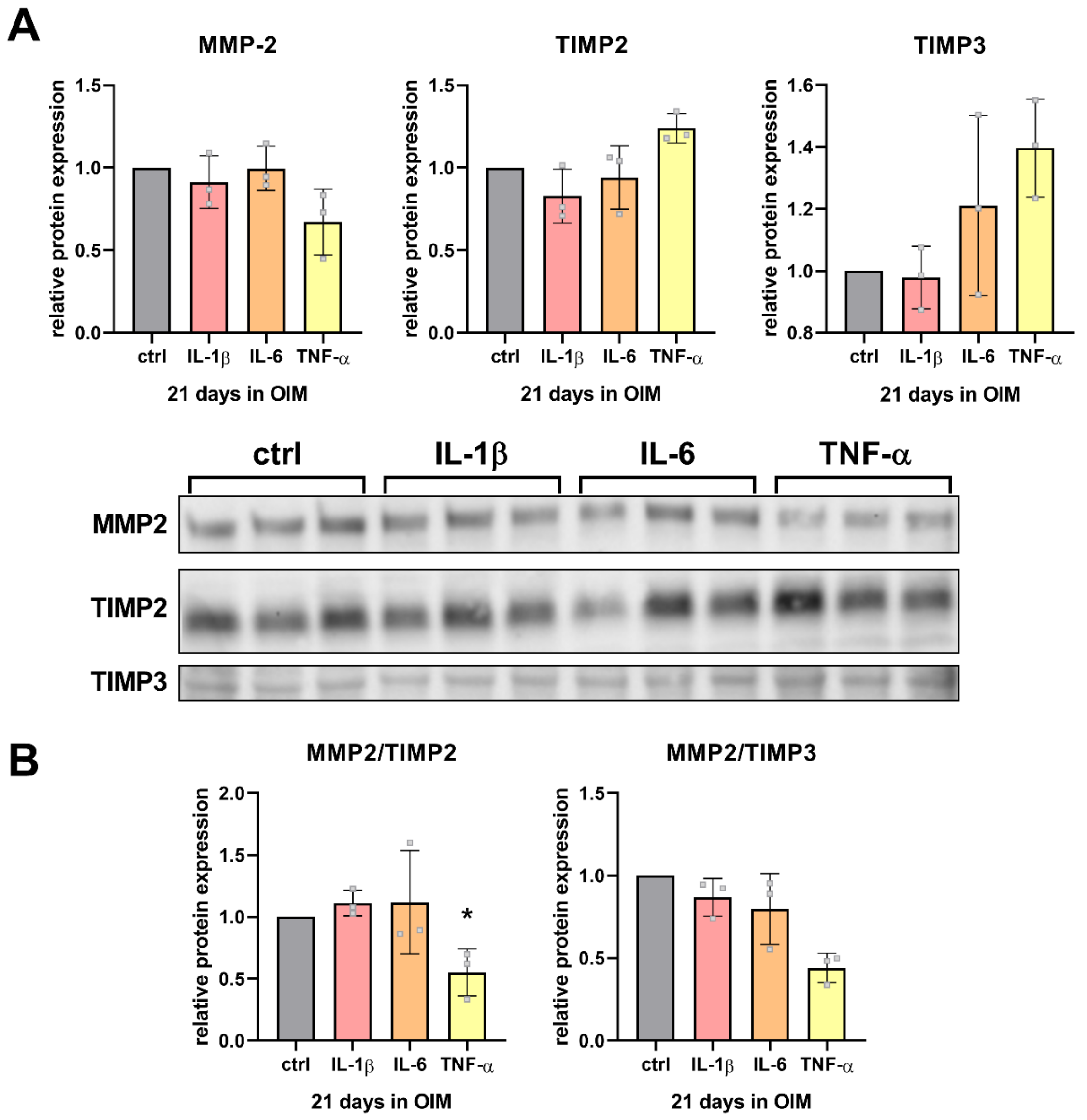

Figure S3: Protein levels of MMP2, TIMP2, and TIMP3 of cytokine-treated hFOB 1.19 cells on day 21. (A) Protein levels of MMP2, TIMP2, and TIMP3 were assessed by Western blotting at day 21 of osteogenesis with cytokine treatment. Protein signals were normalized to total protein (30 µg total protein loaded). (B) The MMP2/TIMP2 and MMP2/TIMP3 ratio was calculated and normalized on the control. Data are shown as mean ± SD (n = 3). Statistical analysis was performed by one-way ANOVA followed by Dunnett's multiple comparisons test ($p \leq 0.05$ (*)).

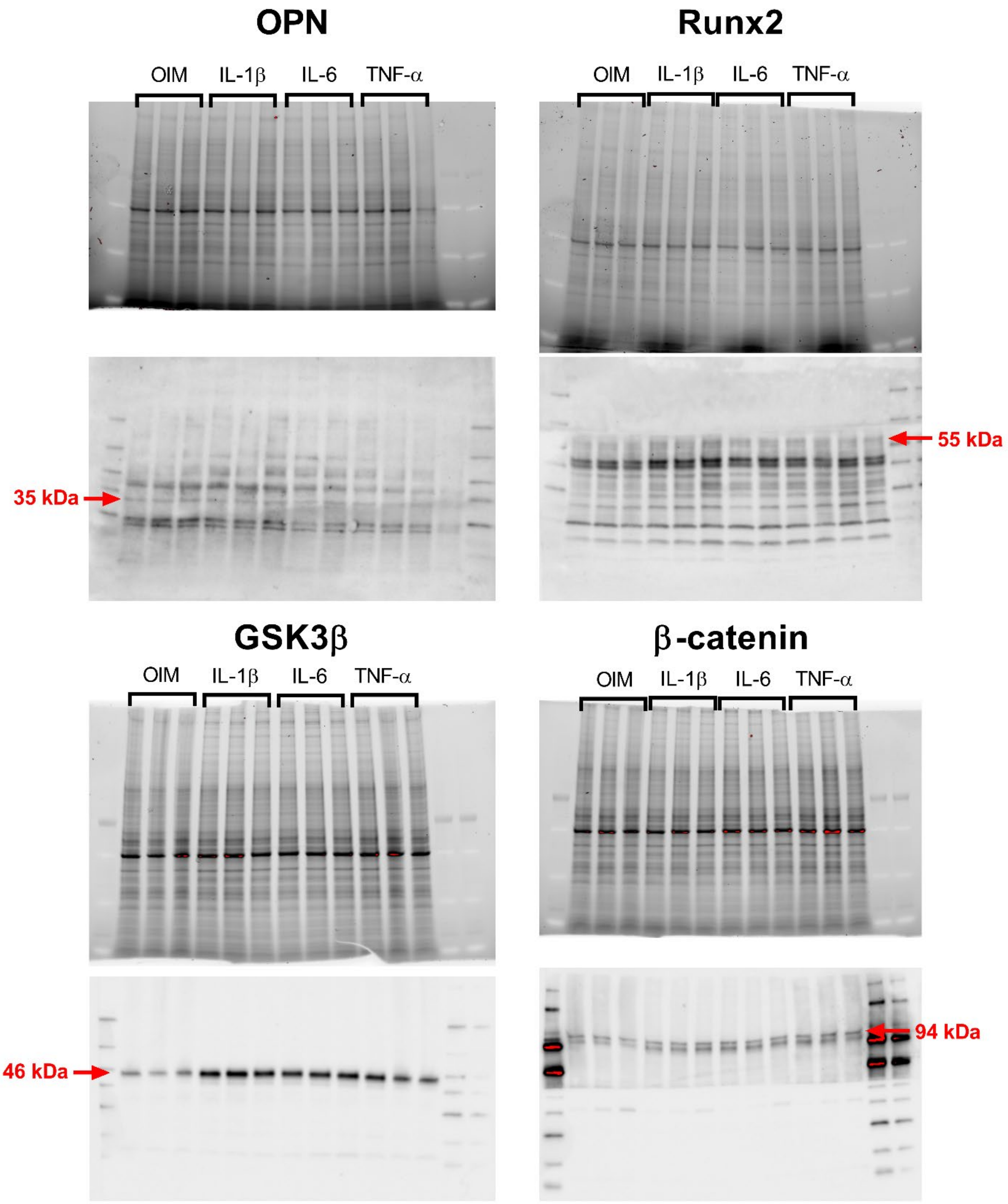

Figure S4: Images of Western blots and corresponding protein gels for OPN, Runx2, GSK3β, and β-catenin protein expression quantification.

Table S1: Patient information of the five OB-donors used for mRNA expression profiles.

Donor	*Age*	*Sex*	*Condition*	*Medication*
OB_1	56	m	coxarthrosis	--
OB_2	88	m	fracture	Phenprocoumon, Allopurinol, Amlodipin, Xipamid, Bicanorm
OB_3	77	f	coxarthrosis	Lercanidipin, Metoprolol, Opipramol, Simvastatin, Candesartan, Torasemid
OB_4	70	m	coxarthrosis	--
OB_5	78	m	fracture	Levothyroxine, Ramipril, Atorvastatin, Metoprolol, Cabergolin, Pantoprazole, Mirtazapine, Valproat, Amlodipin, Hydrocortisone, Tamsulosin, Spasmex, Macrogol, Restex

Table S2: Seeding cell numbers of hFOB 1.19 cells and OBs at different confluences. As OBs and hFOB 1.19 cells vary significantly in size, different cell numbers were seeded to achieve equivalent surface seeding densities.

Experiment	*Confluency*	*Cell Number/Well*	*Cell Culture Plate*
Fig. 1: *Toluidine blue, Oil red O and phalloidin staining*	50 %	hFOB 1.19: $5x10^4$<br>OBs: $2x10^4$	24-well
Fig. 2, 5: *Alizarin Red S staining*	100 %	hFOB 1.19: $1x10^5$<br>OBs: $4x10^4$	24-well
Fig. 5: *ALP assay*	100 %	hFOB 1.19: $1x10^5$	24-well
Fig. 3, 4, 6*: qPCR, WB*	100 %	hFOB 1.19: $7.5x10^5$<br>OBs: $2x10^5$	6-well
Fig. 7: *MTT assay*	100 %	hFOB 1.19: $1x10^5$	24-well
Fig. 7: *BrdU assay*	60 %	hFOB 1.19: $6x10^4$	48-well

Images of Western blots and protein gels for protein expression data shown in the Supplemental Figure S2:

p38 MAPK

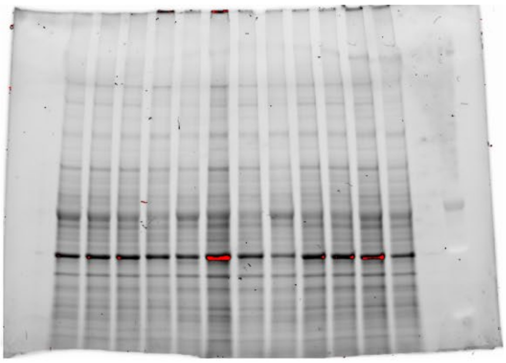

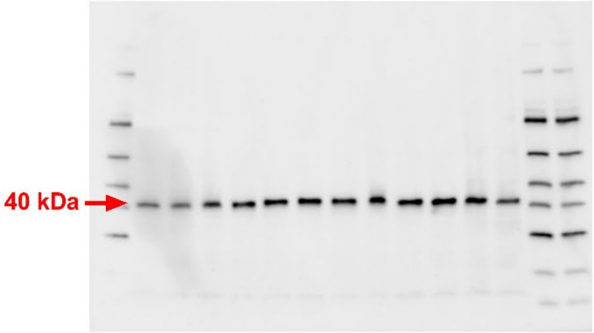

phospho-p38 MAPK

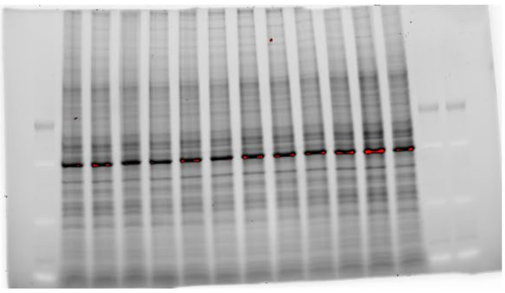

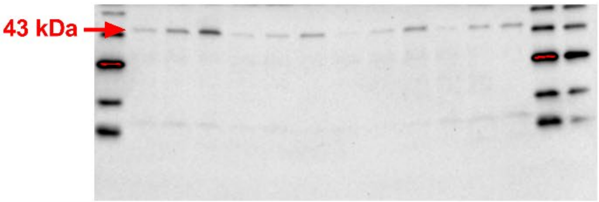

phospho-p42/p44 MAPK

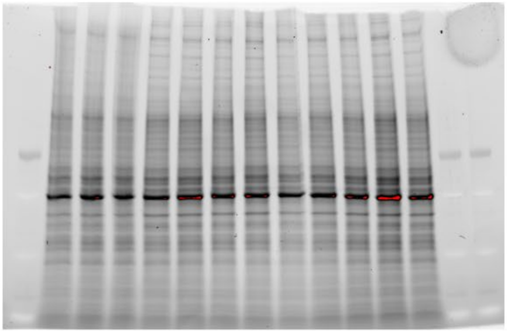

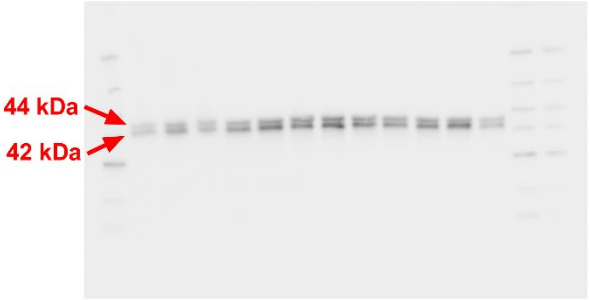

NFκB

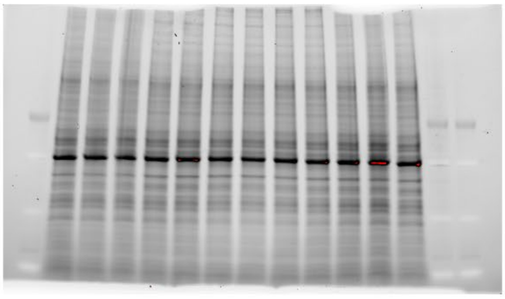

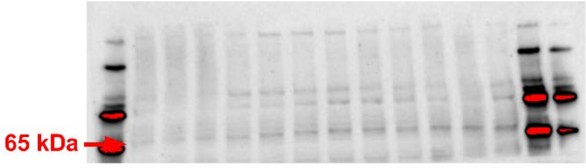

IκBα

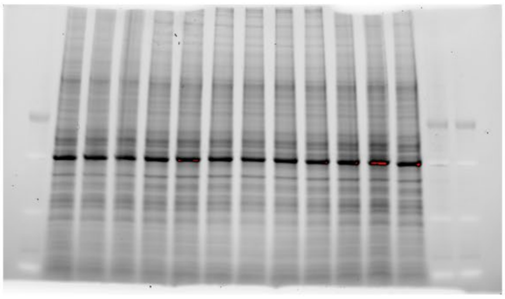

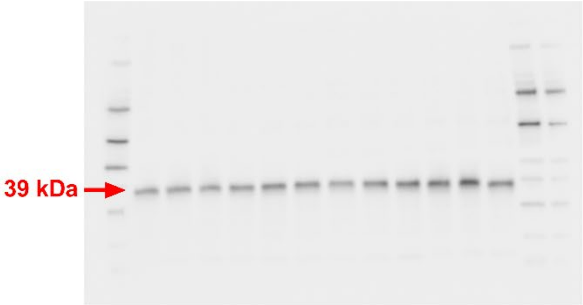

phospho-IκBα

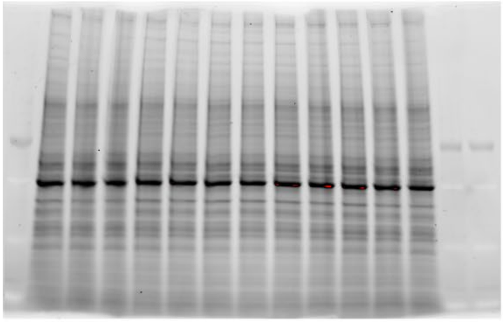

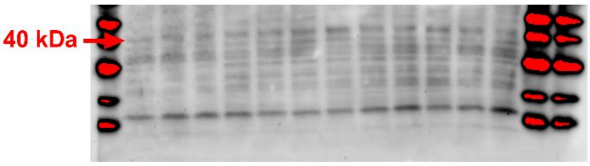

Images of Western blots and protein gels for protein expression data shown in the Supplemental Figure S3:

MMP-2

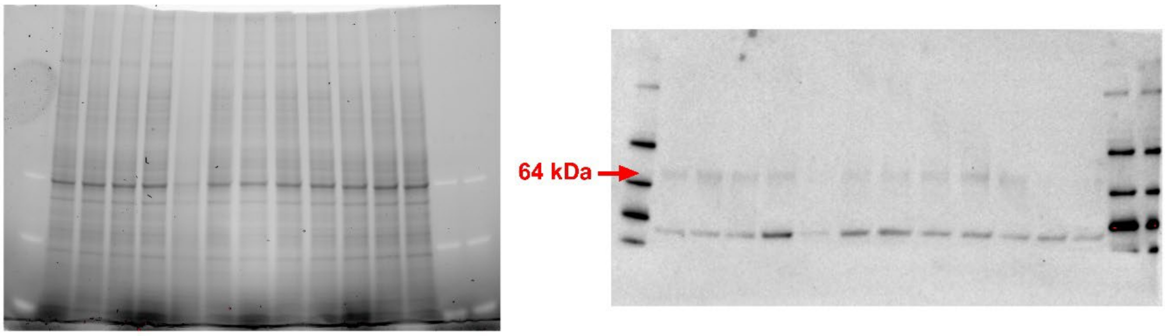

TIMP2

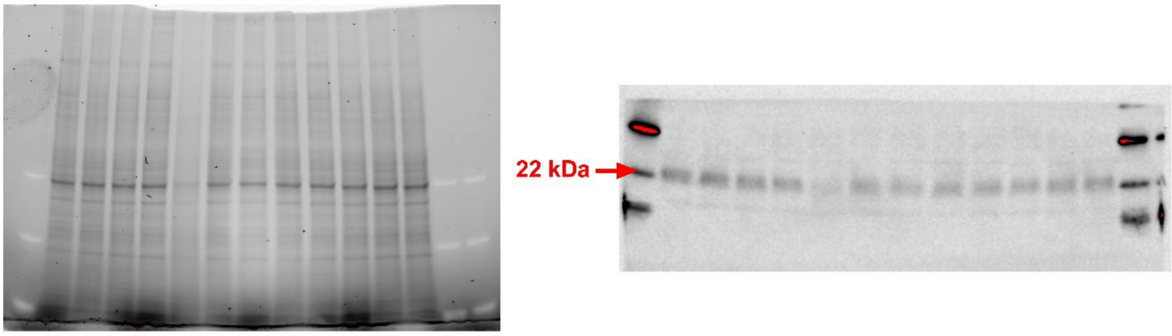

TIMP3

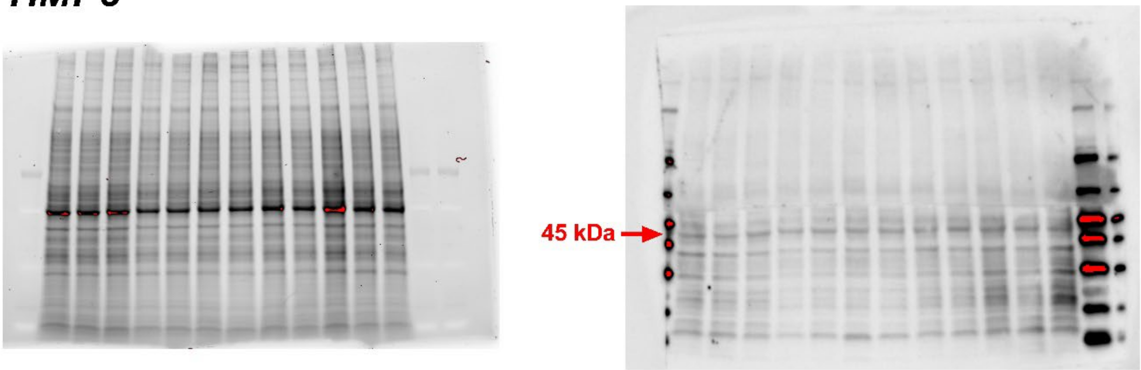

Supplement: Supplementary file 1 [file cells-14-01264-s001.zip › cells-3735366-Supplementary.pdf]
